# Supplementary material for: Transcriptomic Analysis of the CAM Species Kalanchoë fedtschenkoi Under Low- and High-Temperature Regimes
Source: Plants (Basel). 2024 Dec 8;13(23):3444. doi: 10.3390/plants13233444 (PMC11644069; doi:10.3390/plants13233444)

Cellular component

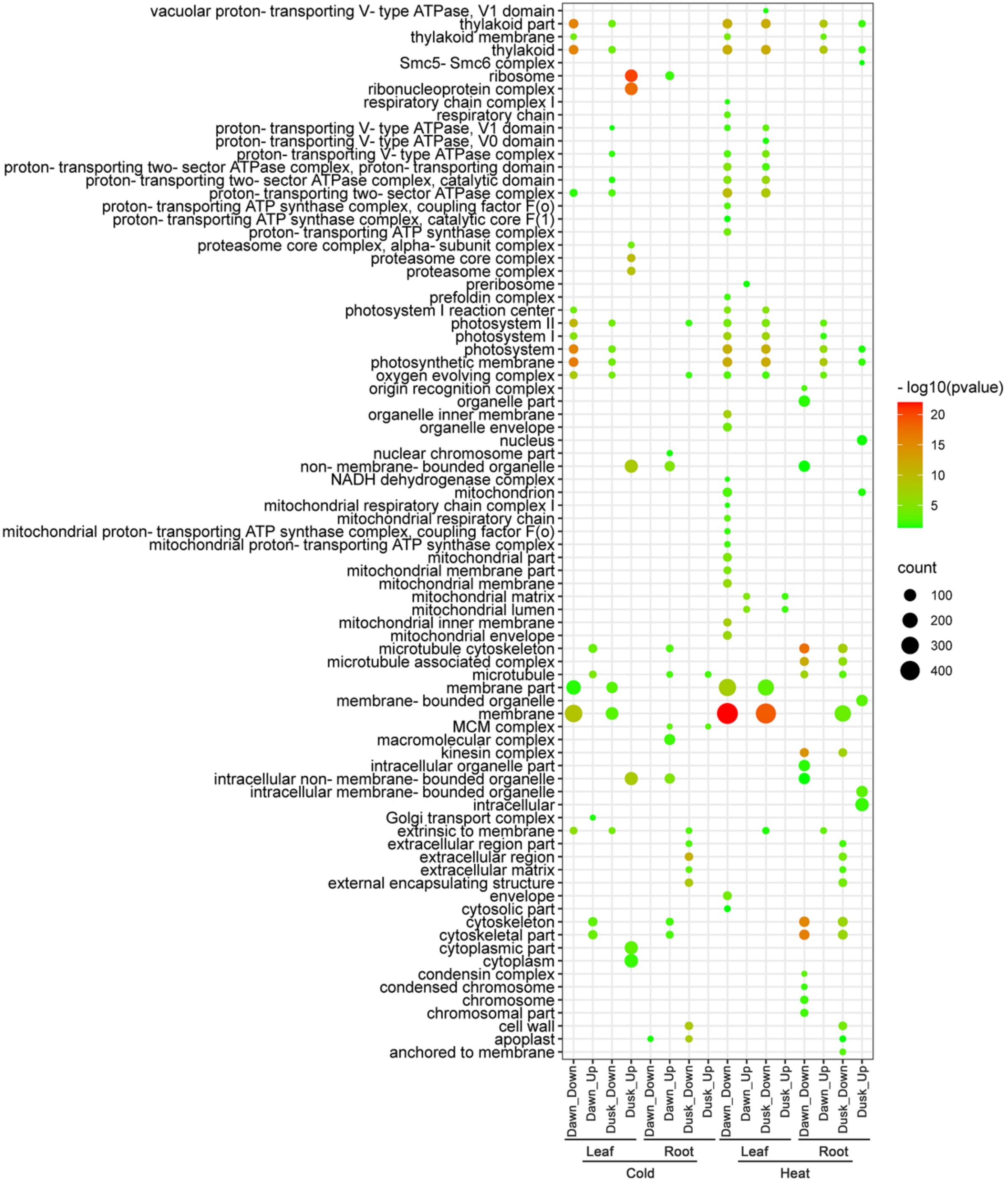

Supplement: Supplementary file 1 [file plants-13-03444-s001.zip › Supplementary Figure S5.pdf]
