# Supplementary figures and images for: Transcriptomic Analysis of the CAM Species Kalanchoë fedtschenkoi Under Low- and High-Temperature Regimes
Source: Plants (Basel). 2024 Dec 8;13(23):3444. doi: 10.3390/plants13233444 (PMC11644069; doi:10.3390/plants13233444)

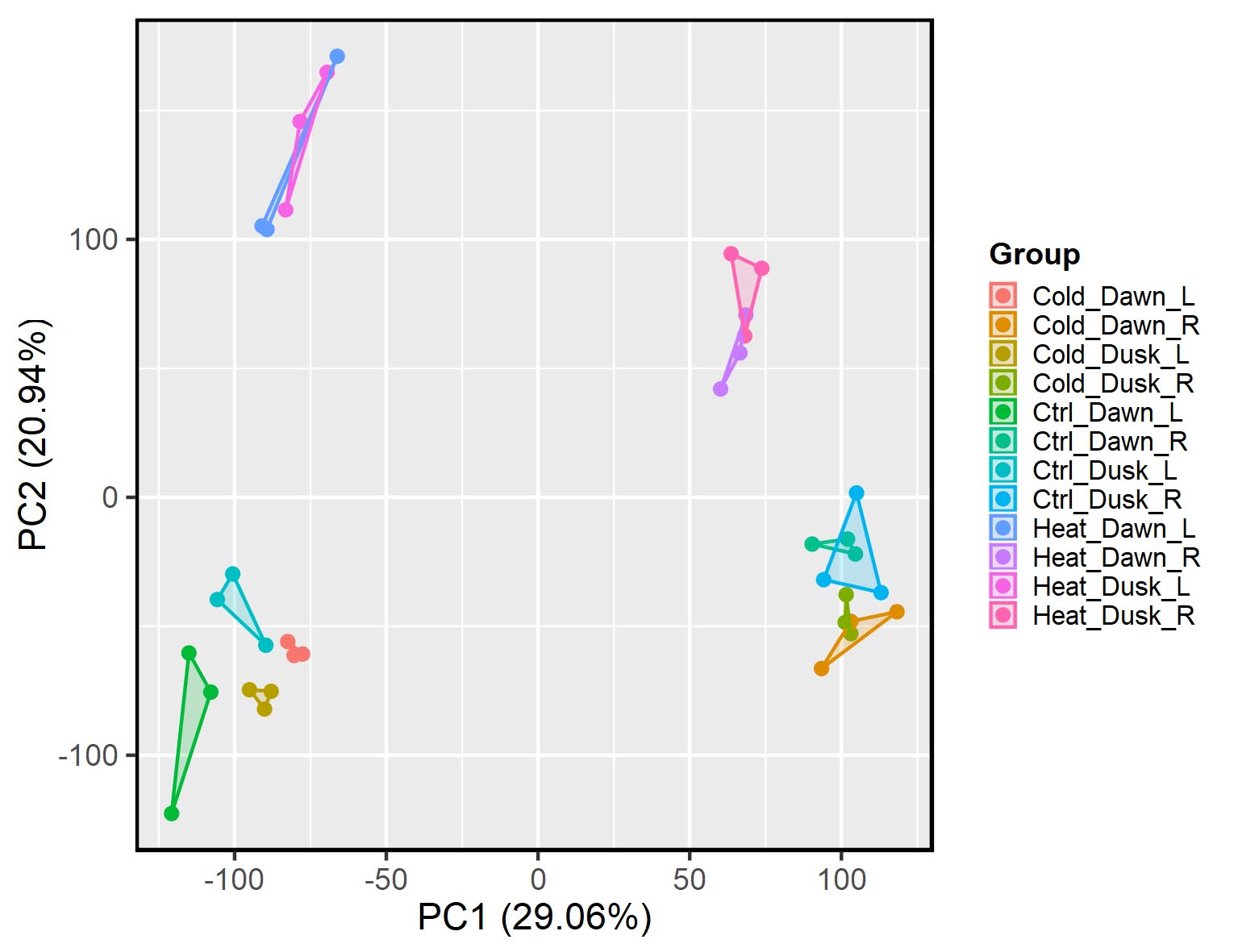

Supplement: Supplementary file 1 [file plants-13-03444-s001.zip › Supplementary Figure S1.jpg]

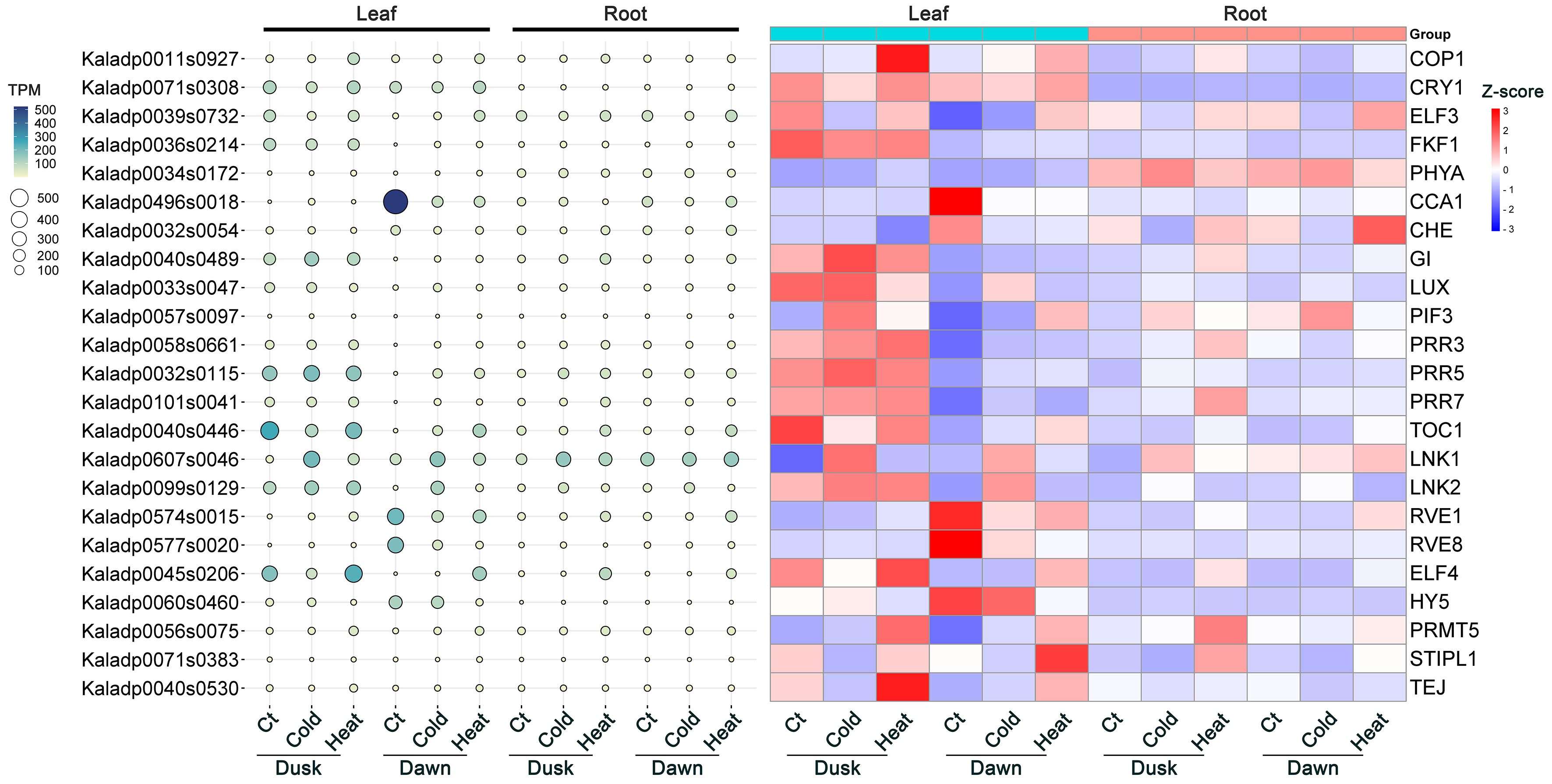

Supplement: Supplementary file 1 [file plants-13-03444-s001.zip › Supplementary Figure S10.jpg]

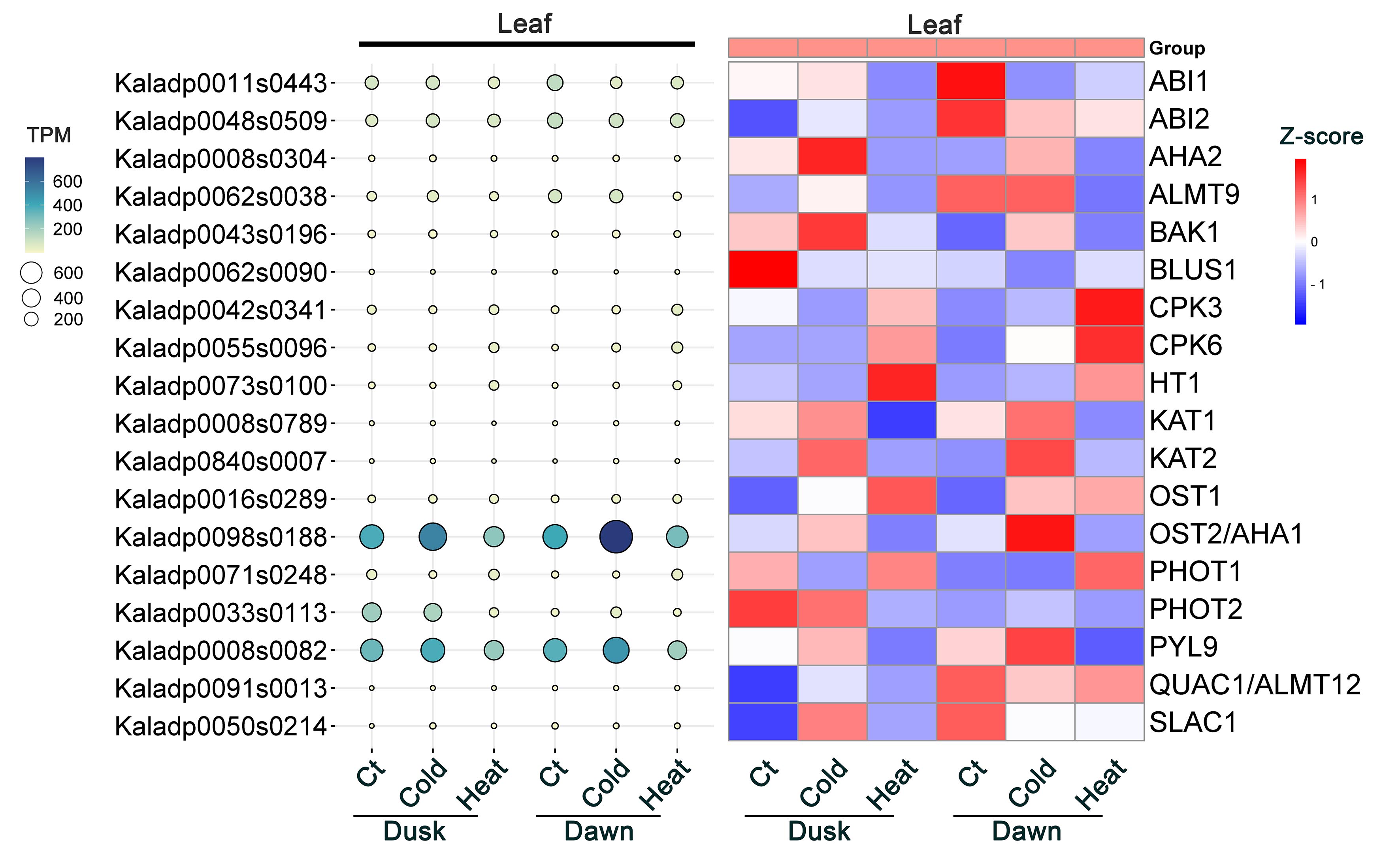

Supplement: Supplementary file 1 [file plants-13-03444-s001.zip › Supplementary Figure S11.jpg]

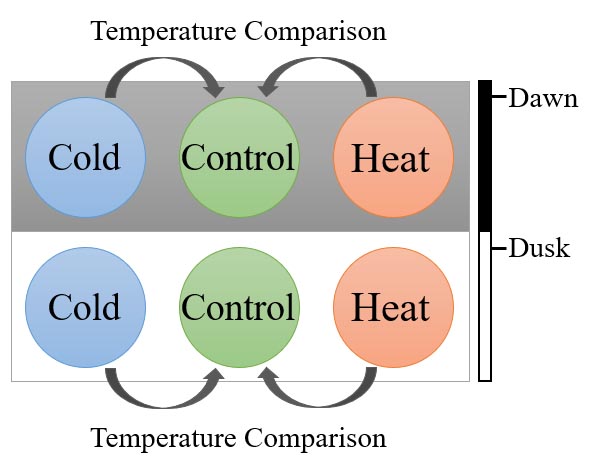

Supplement: Supplementary file 1 [file plants-13-03444-s001.zip › Supplementary Figure S2.jpg]

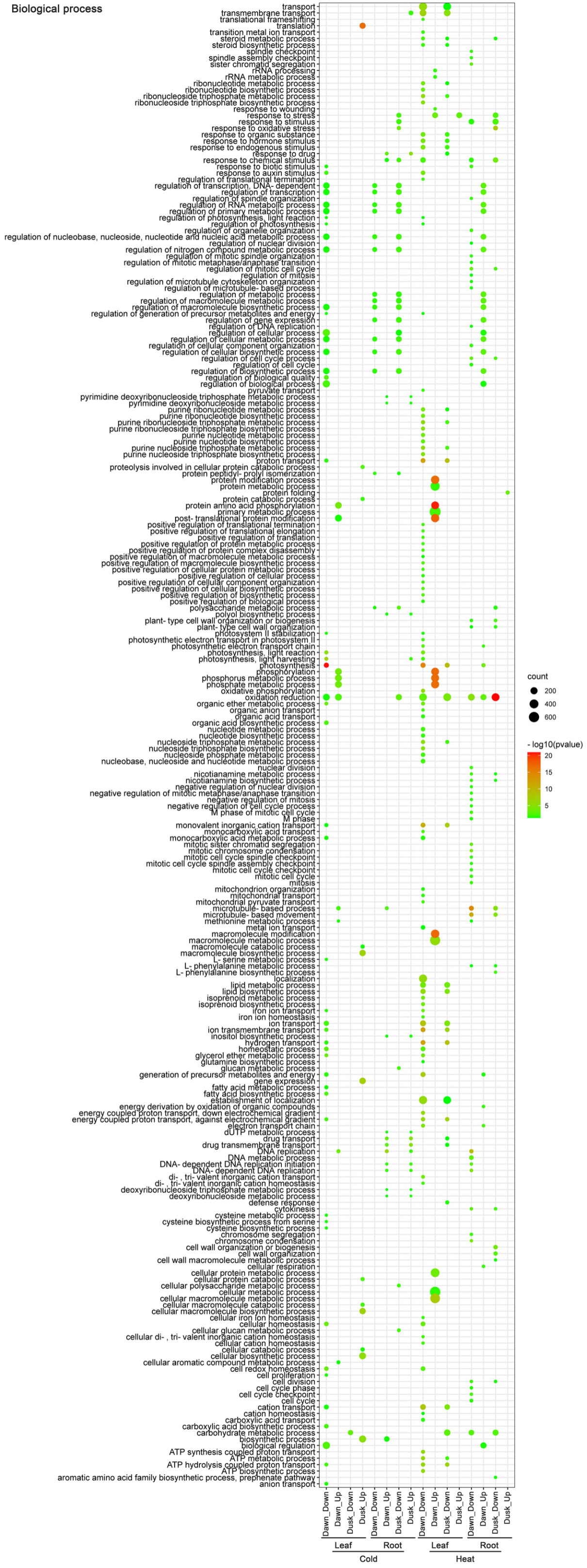

Supplement: Supplementary file 1 [file plants-13-03444-s001.zip › Supplementary Figure S3.pdf]

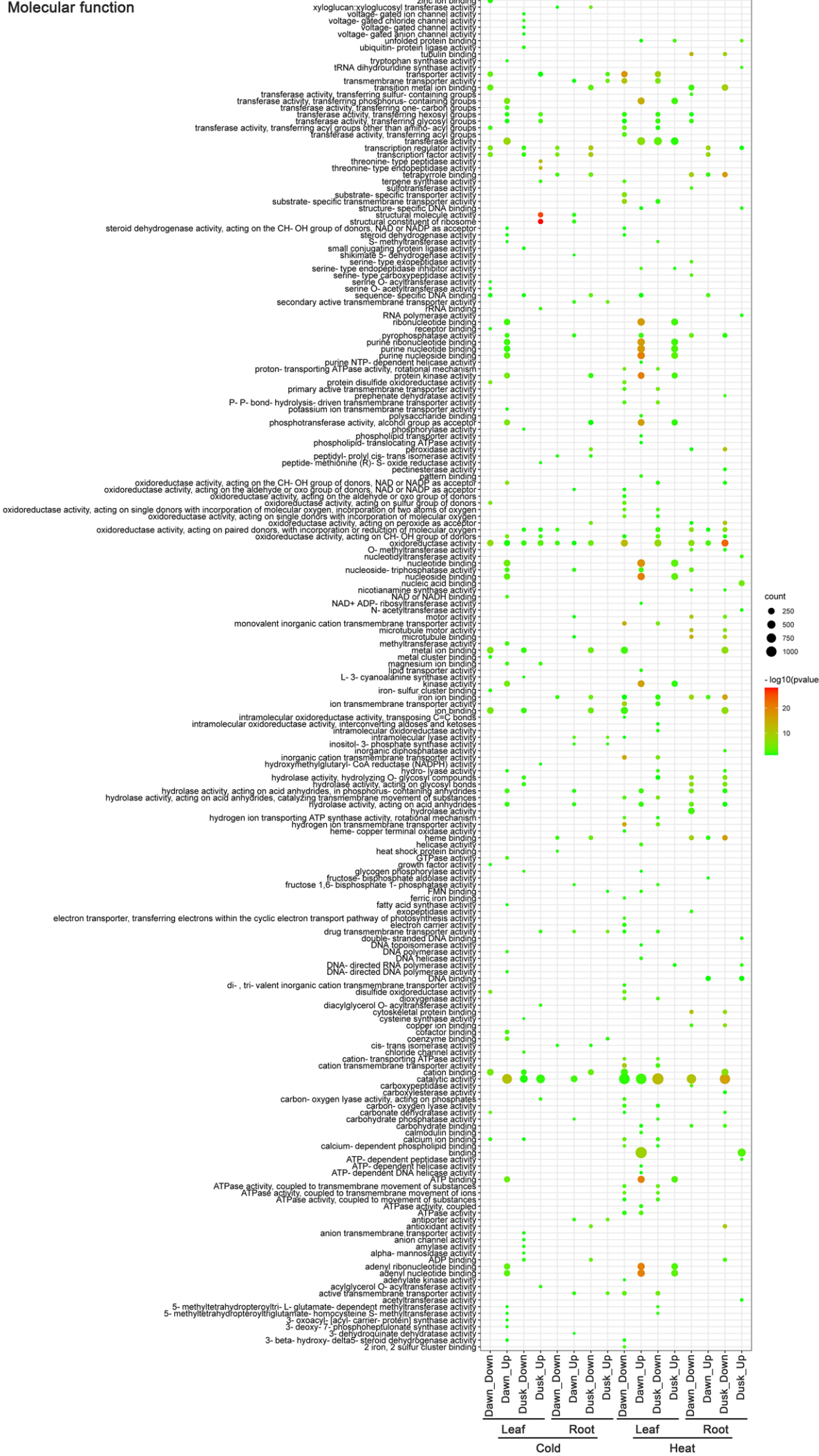

Supplement: Supplementary file 1 [file plants-13-03444-s001.zip › Supplementary Figure S4.pdf]

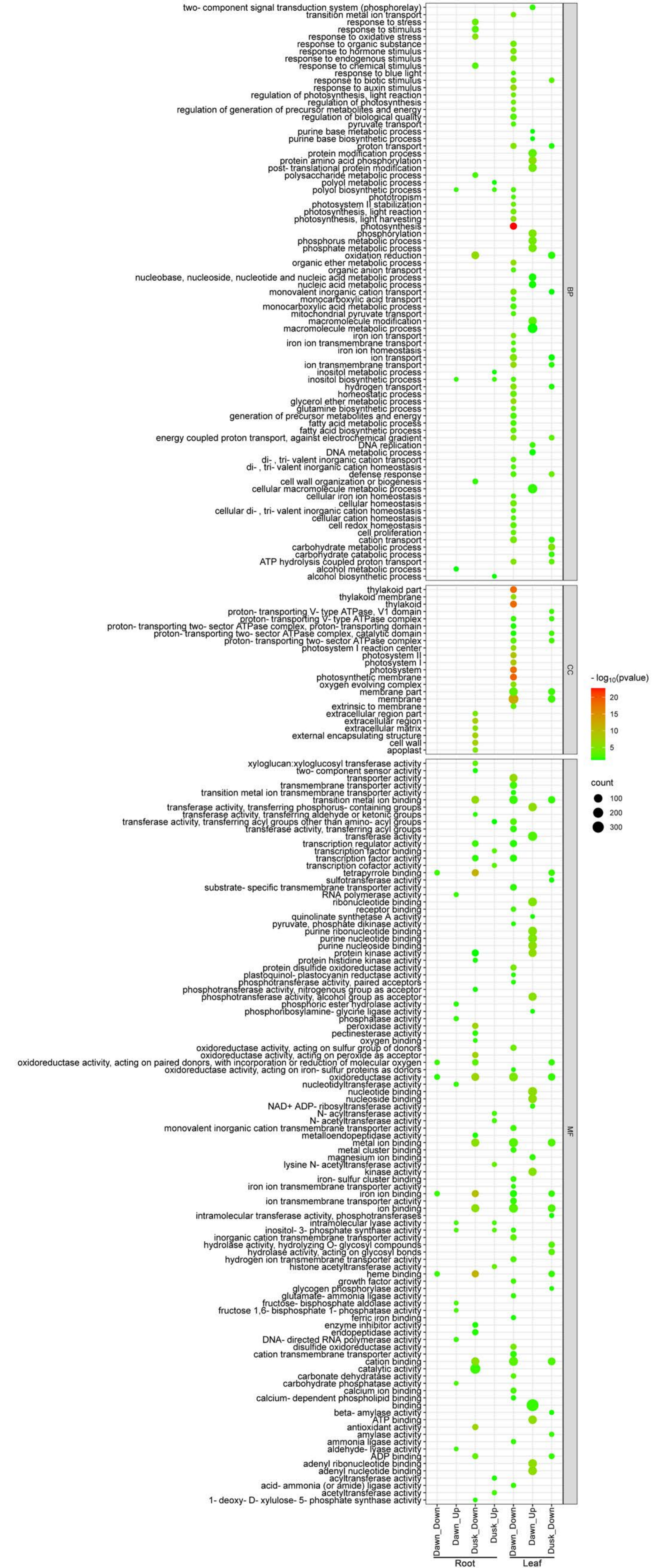

Supplement: Supplementary file 1 [file plants-13-03444-s001.zip › Supplementary Figure S6.pdf]

Biological process

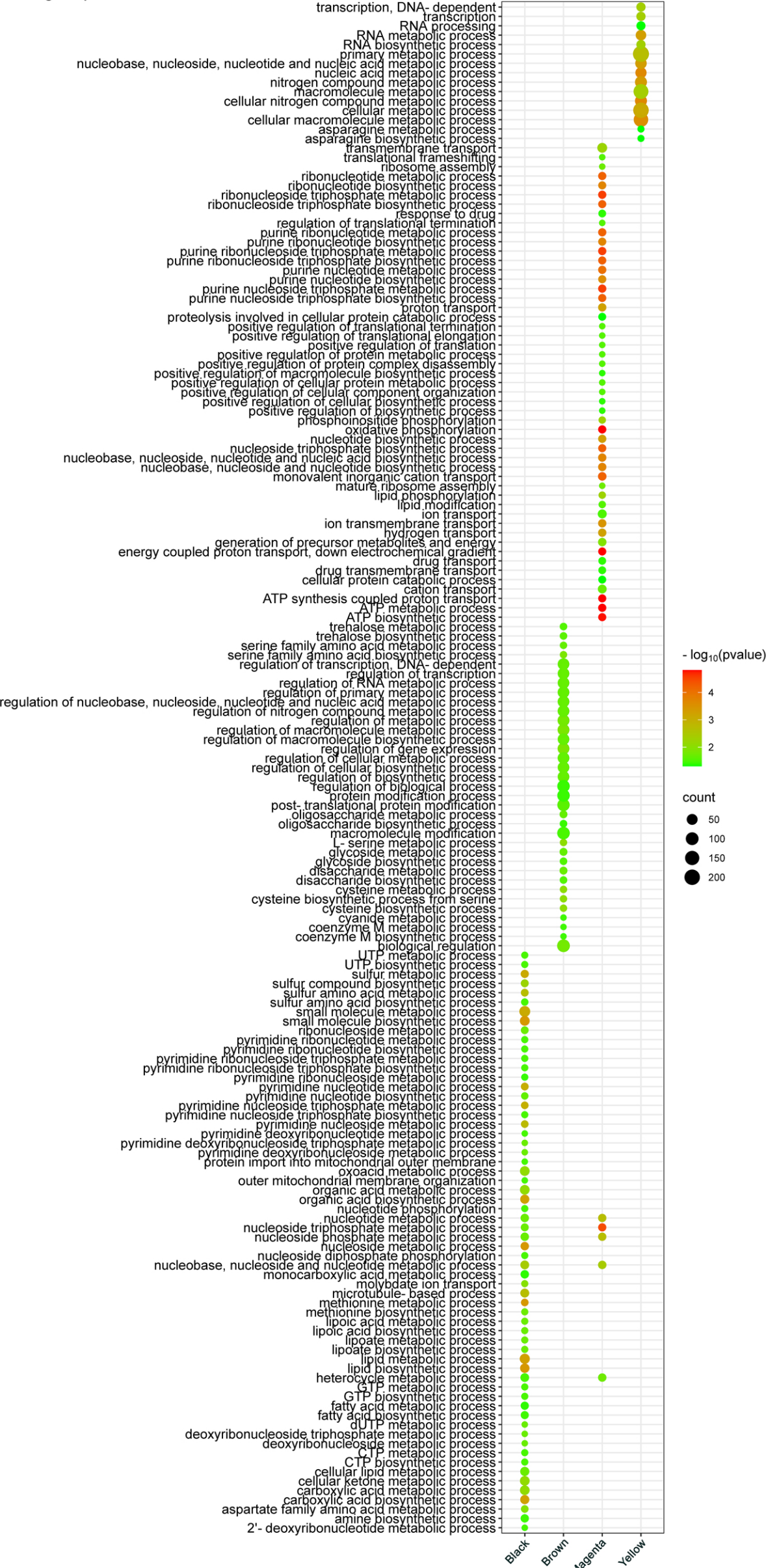

Supplement: Supplementary file 1 [file plants-13-03444-s001.zip › Supplementary Figure S7.pdf]

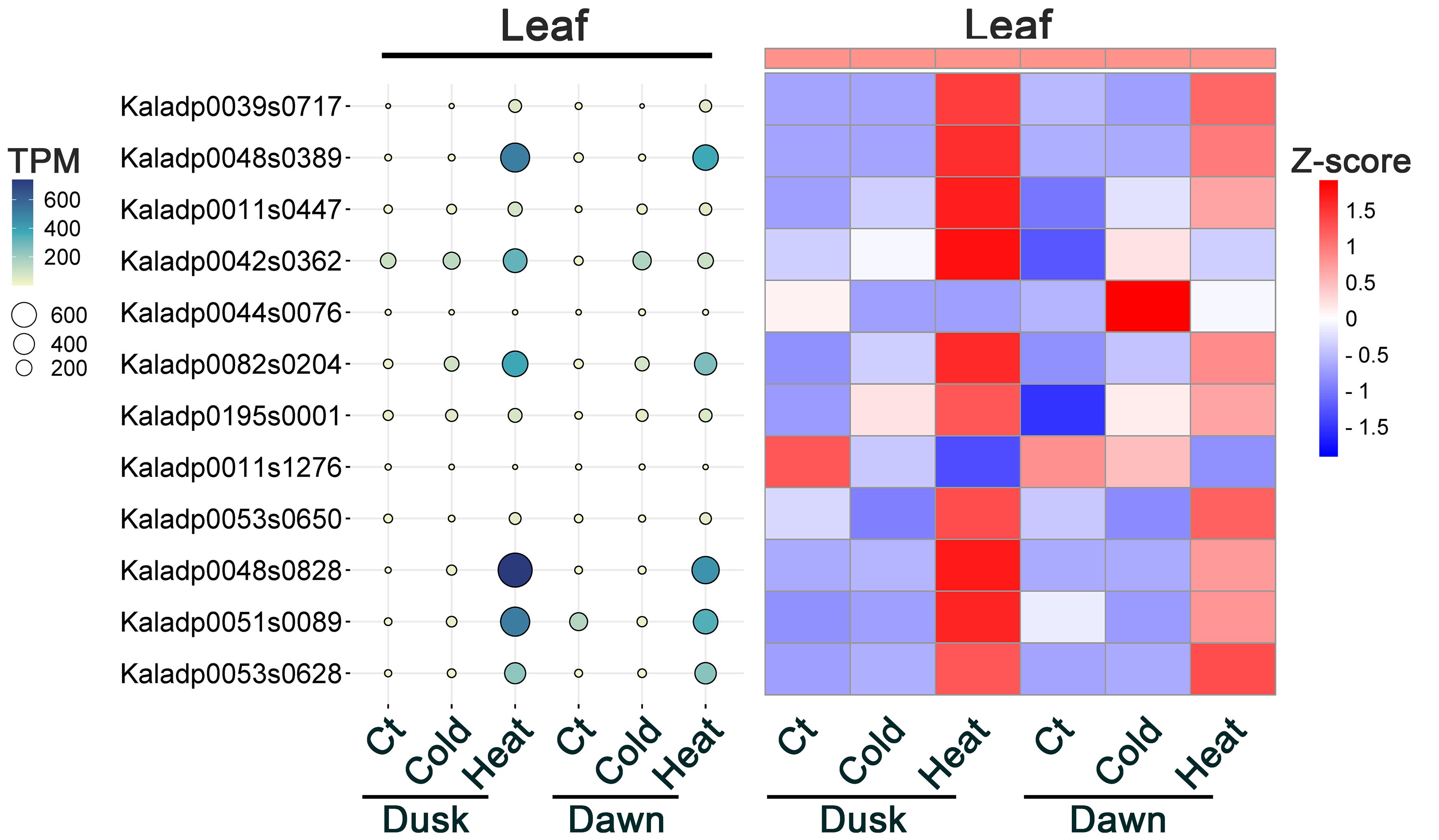

Supplement: Supplementary file 1 [file plants-13-03444-s001.zip › Supplementary Figure S8.jpg]

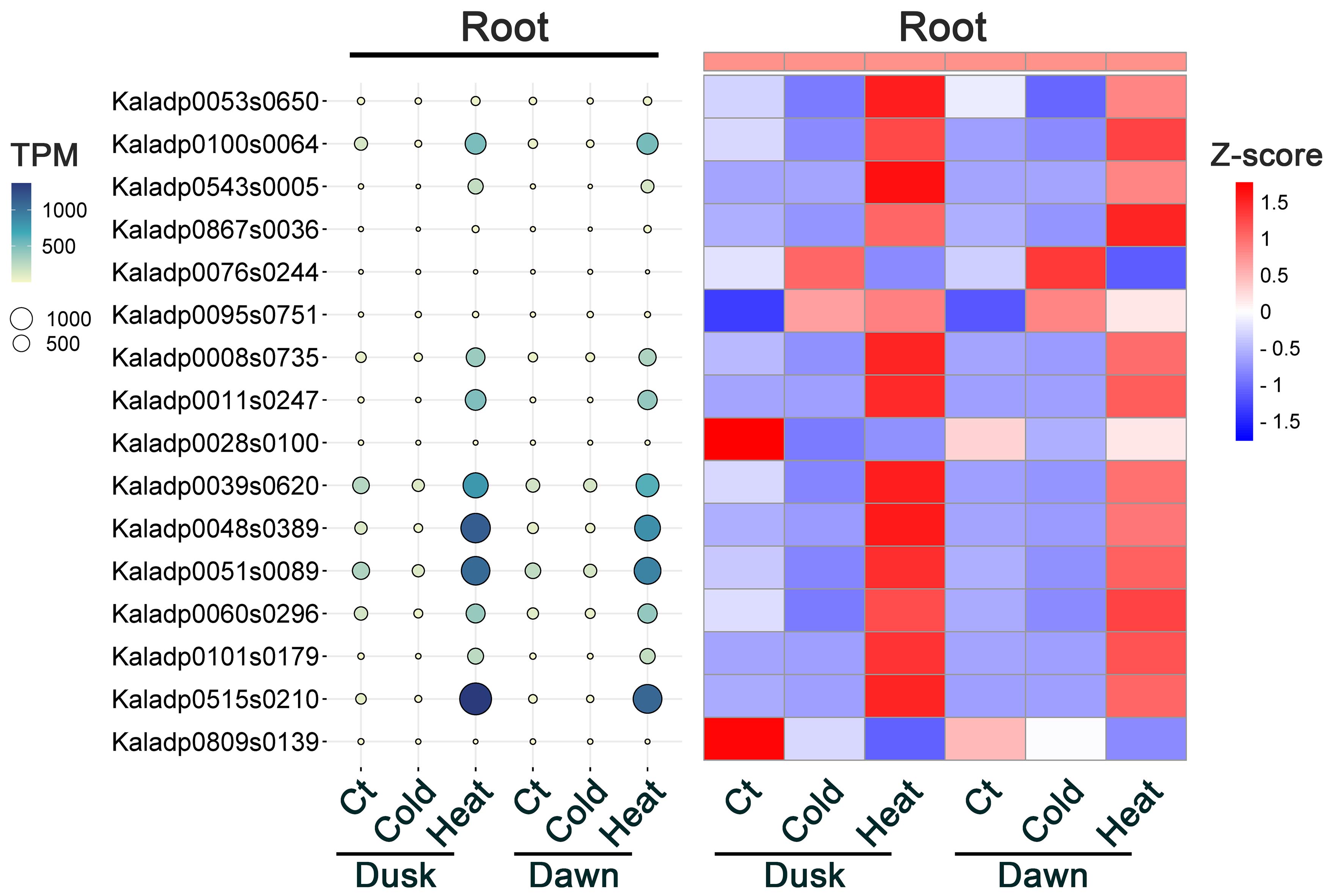

Supplement: Supplementary file 1 [file plants-13-03444-s001.zip › Supplementary Figure S9.jpg]
